# Denosumab: mechanism of action and clinical outcomes

**DOI:** 10.1111/ijcp.12022

**Published:** 2012-09-12

**Authors:** D A Hanley, J D Adachi, A Bell, V Brown

**Affiliations:** 1 Departments of Medicine, Community Health Sciences and Oncology, University of CalgaryCalgary, AL, Canada; 2 Department of Medicine, Michael G. DeGroote School of Medicine, St. Joseph’s Healthcare, McMaster UniversityHamilton, ON, Canada; 3 Department of Family and Community Medicine, University of TorontoToronto, ON, Canada; 4 Department of Family and Community Medicine, University of Toronto, McMaster UniversityHamilton, ON, Canada

## Abstract

**Aims:**

To describe the mechanisms of action of denosumab, a novel antiresorptive agent, contrasting it with other antiresorptive and anabolic osteoporosis treatments.

**Methods:**

Published papers related to the mechanism of action of approved osteoporosis treatments were sought through MEDLINE searches.

**Findings:**

Osteoporotic fractures carry a substantial burden of morbidity and mortality, but pharmacotherapy can prevent such fractures in high-risk individuals. Antiresorptive drugs (e.g. bisphosphonates, oestrogen, denosumab) reduce bone turnover by distinct mechanisms. Denosumab, a recently approved therapy, is a fully human monoclonal antibody that binds the cytokine RANKL (receptor activator of NFκB ligand), an essential factor initiating bone turnover. RANKL inhibition blocks osteoclast maturation, function and survival, thus reducing bone resorption. In contrast, bisphosphonates bind bone mineral, where they are absorbed by mature osteoclasts, inducing osteoclast apoptosis and suppressing resorption. These differences in mechanism influence both the onset and reversibility of treatment.

**Discussion:**

Effective pharmacotherapy is necessary for patients at high risk of fracture. Among the treatment options for postmenopausal osteoporosis, there are significant differences in mechanism and dosing. Denosumab acts by a novel mechanism and is administered twice yearly by subcutaneous injection. Identified by Osteoporosis Canada Clinical Practice Guidelines as a first-line agent for treatment of postmenopausal osteoporosis, denosumab represents an important addition to our treatment options.

**Review criteria**

Studies and review articles related to therapies for postmenopausal osteoporosis were sought via electronic databases and were identified from key references within articles. Search terms and MeSH headings used included *mechanism of action* combined with the word *osteoporosis* and each of the following: *denosumab, antiresorptive*, *bisphosphonate*, *parathyroid hormone* and *RANK ligand*. No formal evaluation of level of evidence was conducted in developing this narrative review.

**Message for the clinic**

Fractures carry a substantial burden of morbidity and mortality, but are preventable by pharmacotherapy in high-risk patients. Mechanistic differences between therapeutics used for postmenopausal osteoporosis have important implications for the timing and reversibility of treatment.

Osteoporosis is a systemic skeletal disease that increases with age and is common among postmenopausal women (1–5). Characterised by reduced bone mineral density (BMD) and weakened bone structure (2,3,6–8), osteoporosis decreases bone resistance to low-energy trauma and increases bone fragility and fracture risk (6,8,9). Almost all pharmacological agents for osteoporosis specifically target the bone resorption component of bone remodelling pathways; they are therefore classified as anticatabolic or antiresorptive agents (e.g. the bisphosphonates etidronate, alendronate, risedronate and zoledronic acid; oestrogen and the selective oestrogen receptor modulator (SERM) raloxifene; salmon calcitonin; and denosumab). The only anabolic agent currently available is teriparatide (7). These treatments reduce the risk of osteoporotic fractures and stabilise or increase bone mass and strength (10).

This article aims to review the mechanisms of action of pharmacological therapies for osteoporosis and to clarify the differences between the bisphosphonates and denosumab, a newly approved antiresorptive agent with a novel mechanism of action (7,11). Denosumab is a fully human monoclonal antibody that binds RANKL, preventing RANKL from activating RANK, its receptor on the osteoclast surface (11). With reduced RANK–RANKL binding, osteoclast formation, function and survival are inhibited, bone resorption decreases and bone mass increases (11–13).

## Findings

### The prevalence of osteoporosis and the care gap

Osteoporotic fractures account for approximately 80% of all fractures occurring in postmenopausal women (14). Based on data from 2000 to 2005, it is estimated that more than 138,000 such fractures occur annually in Canada (15). In Ontario, more than half a million individuals were estimated to have osteoporosis in 2005, leading to approximately 57,000 osteoporosis-related fractures per year, along with $500 million in hospitalisation and long-term care costs (16).

Incidence of osteoporotic hip fracture (approximately 21,000–25,000 per year in Canada) (15,17) is similar to that of breast cancer, heart attack or stroke (15). Such fractures are associated with a 25% risk of death within the following year, with continued elevated mortality in the second year following the event. Vertebral fracture, which is still more common (approximately 37,000 per year), is likewise associated with significantly increased mortality in the first and second year after the event (15). In a prospective study, Papaioannou et al. found that men and women over 50 years of age with hip fractures showed quality-of-life (QoL) deficits, particularly affecting mobility, ambulation and self-care (4). Deficits increased with the number of fractures and were similar to those in other chronic conditions, such as diabetes, arthritis and lung disease (18).

According to a meta-analysis of eight studies, an overall 10% reduction in mortality is achievable with osteoporosis pharmacotherapy; this benefit is clearest for older, frailer individuals at high risk of fracture (19). When used as prescribed, pharmacological agents also offer significant QoL improvement among older women at risk of fracture (20).

Despite abundant evidence of the high burden of mortality and morbidity imposed by osteoporosis in older Canadian women, and despite the clear benefits of pharmacotherapy in higher risk women (14), a care gap remains in the identification and management of this disease. A Quebec-based prospective study, for instance, showed that < 20% of women with incident osteoporotic fractures were prescribed pharmacotherapy for osteoporosis during the 6–8 months following the event (21).

### Osteoporosis and cell biology of the bone

**Figure 1:**
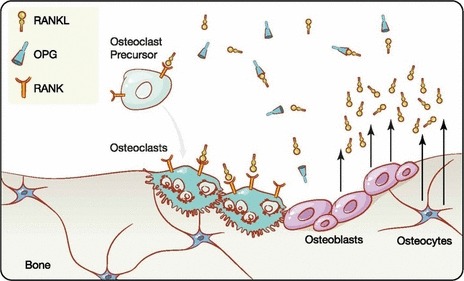
Resorption of old bone matrix and deposition of mineral into new bone are linked. Osteocytes, osteoblasts and osteoclasts are the main cells of the BMU of remodelling bone. BMUs like the one depicted here occur by the millions throughout the skeleton. They carry out the sequential resorption of old bone matrix and deposition/mineralisation of new bone. Osteocytes form a network of interconnected cells occupying lacunae (pits) within the mineralised bone tissue. Osteocytes are derived from osteoblasts (bone-forming cells) that were buried as new bone tissue formed, and they direct bone remodelling in response to mechanical strain and other stimuli. Osteocytes and osteoblasts initiate bone remodelling and start the process of bone resorption by releasing RANKL, which binds to RANK on osteoclasts and osteoclast precursors, activating these cells. Osteoblasts also produce OPG, which suppresses bone turnover. OPG binds to RANKL, preventing it from interacting with RANK. Activation of bone remodelling in a BMU therefore depends on the balance between RANKL and OPG. Adapted from, with permission of John Wiley & Sons, from Denosumab: Mechanism of Action and Clinical Outcomes, Sundeep Khosla, Jennifer J Westendorf, Merry Jo Oursler, 118, 2, 2008; permission conveyed through Copyright Clearance Center Inc.

Normal bone remodelling is modulated by local and systemic regulators (22). Bone resorption and formation are normally in balance, enabling the repair of microdamage, maintenance of calcium homoeostasis and a stable bone mass (23). Bone is continually remodelled by the interaction of osteoclasts (which resorb the existing bone) and osteoblasts (which form new bone matrix). As shown in Figure 1, these two cell types work together with resident bone osteocytes in the basic multicellular units (BMUs) that carry out bone remodelling (1,9,24).

Bone loss and structural damage occur when the extent of bone resorption within a BMU exceeds that of bone formation (negative bone balance) (24). An important cause of negative bone balance is menopause, when falling oestrogen production leads to an increase in RANKL secretion by osteoblasts and osteocytes, in turn increasing activation of osteoclast precursors and mature osteoclasts. Thus, bone resorption and bone remodelling accelerate as ovarian function declines; the increased action of RANKL results in a longer lifespan of osteoclasts and increased rate of bone remodelling in postmenopausal osteoporosis (25,26).

### Classes of osteoporosis medications

**Figure 2:**
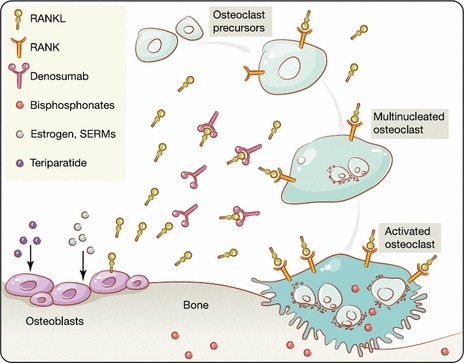
Sites of action for first-line osteoporosis treatments. Teriparatide, a recombinant fragment of parathyroid hormone, stimulates bone formation by increasing osteoblast activity and, to a lesser extent, inhibiting osteoclast recruitment. All other approaches shown here are antiresorptive, reducing bone turnover by targeting osteoclasts. Estrogen replacement therapy and likewise the SERM raloxifene interfere with various osteoblast-derived factors that stimulate osteoclasts (e.g. IGF1, TGF-β and TNF-α). Denosumab binds the cytokine RANKL, preventing it from binding its receptor, RANK. Like OPG (see Figure 1), denosumab prevents maturation of osteoclast precursors and promotes apoptosis of mature, multinucleated osteoclasts. Bisphosphonates bind to bone mineral and are taken up by osteoclasts, causing them to undergo apoptosis or have reduced resorptive capacity. When osteoclast number and activity decline, bone formation eventually slows to maintain a balance of bone resorption and formation

There are two main pharmacological approaches to osteoporosis: anabolic therapy, which stimulates new bone formation (27); and anticatabolic or antiresorptive therapy, which decreases bone resorption and/or inhibits bone turnover (14). Molecular and cellular targets of anabolic and antiresorptive treatments are shown in Figure 2.

#### Anabolic therapy

Teriparatide, a recombinant fragment of human parathyroid hormone (PTH 1-34), is the sole anabolic agent approved for treating postmenopausal osteoporosis (14).

The anabolic effect of teriparatide is a common point of confusion among practitioners and merits some comment. Primary hyperparathyroidism features continuous excessive parathyroid hormone secretion and is associated with hypercalcaemia and bone fragility (28). However, PTH (and likewise teriparatide) is short-lived in circulation, and repeated acute exposure (pulsatile dosing) induces an unique physiological response, leading to an increase in osteoblast number and function and increased bone formation. Teriparatide is injected subcutaneously on a once-daily schedule to stimulate new bone formation (29,30).

There is strong evidence that this agent can be used to prevent vertebral and non-vertebral fractures, but insufficient evidence regarding hip fractures (Table 1) (14).

**Table 1:** First-line therapies for osteoporosis, as identified by the 2010 Osteoporosis Canada Clinical Practice Guidelines

			Statistically significant relative fracture risk reductions vs. control				
					
Medication (reference)	Indication(s) in PMO	Pivotal trial name (reference)	Vertebral	Non-vertebral	Hip	Administration	Dose
Alendronate (49)	Treatment and prevention of osteoporosis in postmenopausal women	FIT I (50)	√	X	√	Oral	5 mg daily for prevention of osteoporosis; 10 mg daily (alternatively 70 mg once weekly) for treatment
		FIT II (51)	√	X	X		
Risedronate (52)	Treatment and prevention of osteoporosis in postmenopausal women	VERT NA (53)	√	√	NR	Oral	5 mg daily (alternatively 35 mg once weekly or 150 mg once monthly) for prevention and treatment
		HIPS (54)	NR	NR	√		
Zoledronic acid (55)	Treatment of osteoporosis in postmenopausal women, to reduce the incidence of hip, vertebral and non-vertebral fractures; prevention of postmenopausal osteoporosis in women with osteopenia	HORIZON (56)	√	√	√	Intravenous	5 mg as single 15–30 min infusion once yearly for treatment
Denosumab (11)	Treatment of postmenopausal women with osteoporosis at high risk for fracture, defined as a history of osteoporotic fracture, or multiple risk factors for fracture; or patients who have failed or are intolerant to other available osteoporosis therapy	FREEDOM (48)	√	√	√	Subcutaneous	60 mg every 6 months
Raloxifene (37)	Treatment and prevention of osteoporosis in postmenopausal women	MORE (36)	√	X	X	Oral	60 mg daily
Estrogen replacement therapy^*^	Varies by formulation	WHI (57)	√	NR	√	Oral or transdermal	Daily
Teriparatide (58)	Treatment of postmenopausal women with severe osteoporosis who are at high risk of fracture or who have failed or are intolerant to previous osteoporosis therapy	FPT (59)	√	√	NR	Subcutaneous	20 mcg daily

√, Significant benefit (p < 0.05) shown in pivotal trial; X, no significant effect; NR, not reported; PMO, postmenopausal osteoporosis. ^*^For menopausal women requiring treatment of osteoporosis in combination with treatment for vasomotor symptoms.

#### Antiresorptive therapies

A variety of therapeutic approaches fall under the general category of antiresorptive treatment. Common to all of these approaches, bone turnover occurs more slowly (24), allowing for more extensive mineralisation. Antiresorptive treatments primarily target osteoclasts, reducing their lifespan or activity; they may have secondary effects on osteoblasts or osteocytes (31). This is in contrast to PTH, which acts primarily on osteoblasts, increasing calcium and phosphate incorporation into the bone matrix (32).

Of the antiresorptive therapies, bisphosphonates are the most widely used for patients with osteoporosis. Bisphosphonates have been shown to prevent vertebral, hip and non-vertebral fractures, as well as decrease the mortality rate among those at high fracture risk (10,14,19,33).

Bisphosphonates all induce osteoclast apoptosis; bone-associated osteoclasts that do survive may remain in the bone, but with reduced resorptive activity (31,34). Only three bisphosphonates are currently identified by Osteoporosis Canada (OC) Clinical Practice Guidelines (14) as first-line treatment options: alendronate, risedronate and zoledronic acid. All three are nitrogen-containing bisphosphonates (34), which target a specific metabolic enzyme, farnesyl pyrophosphate synthase (FPPS), preventing the normal modification of intracellular proteins required for osteoclast function and survival (23,31,34,35). All three of these bisphosphonates offer significant reduction in fracture risk at vertebral, hip or non-vertebral sites in postmenopausal women with osteoporosis (14,15). As shown in Table 1, there are some differences among them regarding dose and administration.

In addition to the bisphosphonates, raloxifene is also effective in preventing vertebral fractures (14,36,37). Raloxifene mimics the effect of oestrogen in the bone, but it does not stimulate breast and uterine tissues (38). Hormone therapy, using oestrogen or oestrogen–progesterone formulations, can prevent or reverse the effects of menopause, including the excess bone resorption seen in postmenopausal osteoporosis (9,14,39,40). Oestrogen, used alone, can reduce the incidence of vertebral and hip fractures (10). The OC Clinical Practice Guidelines cite both raloxifene and hormone replacement therapy as first-line therapies for postmenopausal osteoporosis; raloxifene for prevention of vertebral fractures, and hormone therapy for women requiring treatment of osteoporosis in combination with treatment for vasomotor symptoms (14).

Denosumab is the newest antiresorptive agent, with a novel mechanism of action (41). Briefly, denosumab is a fully human monoclonal antibody that inhibits RANKL and helps regulate turnover in healthy bone. Denosumab binds with high specificity and affinity to the cytokine RANKL, inhibiting its action; as a result, osteoclast recruitment, maturation and action are inhibited, and bone resorption slows. Denosumab is indicated for postmenopausal women with osteoporosis at high risk of fracture, or for patients who have failed or are intolerant to other available osteoporosis therapies (11). OC Clinical Practice Guidelines identify denosumab as a first-line option for preventing vertebral, hip and non-vertebral fractures (3,14).

### Denosumab and the bisphosphonates: similarities and differences

Both denosumab and the bisphosphonates specifically target osteoclasts; their effects on osteoblasts are largely indirect, because of the coupling of resorption and bone formation within the BMU (31).

One key to understanding the difference between these antiresorptive agents is their disposition within the body. As shown in Table 2, bisphosphonates have a strong affinity for bone and become embedded in the bone mineral, where they remain until released during bone resorption. Although bisphosphonates ordinarily do not cross cell membranes, they will do so in the acidic environment that osteoclasts create as they resorb the bone matrix, hence their specificity in targeting this cell type.

**Table 2:** Key differences between and bisphosphonates and denosumab

Feature	Bisphosphonates	Denosumab
Molecular target	Cellular metabolic enzymes; for the nitrogen-containing bisphosphonates, the key target is FPPS, an enzyme needed for modification (prenylation) of proteins	Binds with high affinity and specificity to circulating RANKL
Structure	Small-molecule drugs	Monoclonal antibody
Site of action	Tightly bound to mineral in the bone matrix; internalised by osteoclasts	Extracellular milieu; does not associate with bone tissue
Specific effect on osteoclasts?	Yes; needs to be taken up from bone matrix into osteoclast cytoplasm during bone resorption	Yes; affects osteoclasts and their precursors, which express the RANK protein
Effect on osteoclast lineage	Induce apoptosis; bone-associated osteoclasts that survive may remain in the bone, but with reduced resorptive activity	Inhibits osteoclast formation, function, and survival
Onset of action and reversibility of effect	Depends on type of bisphosphonate and length of treatment; slow offset of action	Rapid onset of action; fully reversible and relatively rapid offset of action
Clearance	Release from bone matrix depends on bone turnover; may remain in bone over weeks to years. Released bisphosphonates are cleared by the kidney	Cleared by the reticuloendothelial system with half-life of ∼ 26 days

Modified from Baron et al., 2011 (31). FPPS, farnesyl pyrophosphate synthase.

Clearance of bisphosphonates from the circulation is via renal excretion or adsorption to bone mineral. The initial clearance of a dose of bisphosphonate is rapid, but bone-associated drug must first be released by osteoclast-mediated bone resorption, and removal may extend over a period of weeks to years. There is also significant recycling of bisphosphonates in bone, resulting in retention of measurable amounts for several years (35).

Among the antiresorptive and anabolic therapies for osteoporosis, only the bisphosphonates bind bone matrix, influencing both their onset and offset of action (31). Bisphosphonates differ with respect to their affinity for the bone matrix: zoledronic acid binds more tightly than alendronate, which binds more tightly than risedronate. These biochemical differences may affect the clearance of the bisphosphonate, both immediately after dosing and in the longer term, when bone-associated drug is released by osteoclast action. Skeletal uptake is more efficient for zoledronic acid, relative to the others. Likewise, the duration of action of zoledronic acid and alendronate appear to be greater than that of risedronate, perhaps because they are more efficiently recycled into the bone once they have been released. Thus, differences in bone affinity can influence the required dosing of the bisphosphonates and the reversibility of their effects (34).

In contrast to the bisphosphonates, denosumab (like the other first-line therapeutics) does not become embedded within bone tissue. Rather, by binding to RANKL in the extracellular fluid and circulation, denosumab inhibits osteoclast formation, function and survival (1,7,12,22,31,41,42). As an antibody, denosumab is thought to be cleared from the bloodstream through the reticuloendothelial system, with a half-life of approximately 26 days, and it does not appear to induce the formation of neutralising antibodies (11).

**Figure 3:**
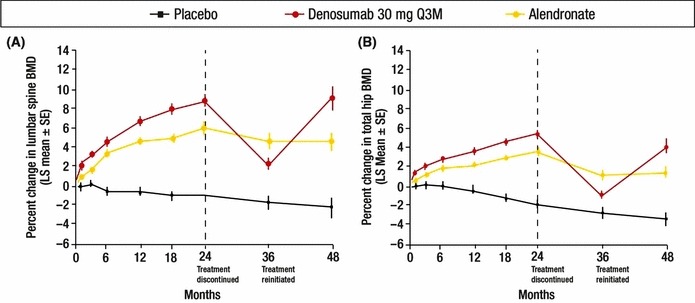
Effect of denosumab treatment discontinuation and reinitiation on bone mineral density in the lumbar spine (A) and total hip (B). Subjects were randomised to denosumab 30 mg Q3M or placebo twice yearly or, on an open-label basis, to alendronate once weekly. Denosumab subjects were transitioned to placebo at Month 24, with their last active treatment at Month 21; they reinitiated active treatment at Month 36 with the 60 mg Q6M dose. Alendronate subjects discontinued treatment at Month 24 and were observed until Month 48. Denosumab’s effects were fully reversible at the hip and lumbar spine and were restored upon retreatment. Placebo-treated patients showed a consistent loss of BMD throughout the study period, affecting both sites. Patients treated with alendronate who subsequently stopped treatment showed little change in BMD at the lumbar spine, but larger decreases in hip BMD. Adapted from Miller et al., 2008 (22). Reprinted from Denosumab: Mechanism of action and clinical outcomes, 43, 2, Paul D. Miller, Michael A. Bolognese, E. Michael Lewiecki, Michael R. McClung, Beiying Ding, Matthew Austin, Yu Liu, Javier San Martin, for the AMG 162 Bone Loss Study Group, 222–229., 2008, with permission from Elsevier.

The bone resorption marker CTx (a fragment of degraded bone collagen protein) declines dramatically following a single 60 mg dose of denosumab, but the effect is reversible. Bone turnover markers return to pretreatment levels within 9 months of treatment cessation (11). Although BMD at various skeletal sites declines to pretreatment levels under these circumstances, it remains higher than in women who received no antiresorptive treatment (13,22). Furthermore, as seen in Figure 3, the BMD lost following treatment cessation can be rapidly restored when treatment is reinitiated (13). However, in the absence of safety concerns arising from ongoing long-term studies (43), patients on denosumab should be encouraged to maintain a regular schedule of injections.

In head-to-head studies (44,45), both alendronate and denosumab led to significant improvement in total BMD, albeit with significantly greater gains in patients on denosumab. However, a high-resolution peripheral quantitative computed tomography (HR-pQCT) study of bone at the distal radius shows that the drugs differ substantially in their effects in cortical bone (45). At the distal one-third radius, treatment over 12 months with denosumab led to an increase in BMD of 1.1%, which was significantly greater than the 0.6% increase with alendronate (p = 0.0001) (44). This increase in cortical bone mass with denosumab is consistent with other findings on cortical BMD and micro-architecture; for each of these measures, benefits with denosumab were significantly greater than with alendronate over the course of 2–3 years [Ref. (45); reviewed in Ref. (31)]. Whether this difference in surrogate markers will translate to fewer wrist fractures remains uncertain.

Interim analysis of ongoing long-term studies suggests that bone density gains with 5 years of denosumab do not plateau (43), as has been seen with other antiresorptive therapies such as zoledronic acid (46). The reason for the apparent continuing rise in bone density with denosumab is not certain. It may result from the recovery of bone remodelling capability at the end of each 6-month cycle of therapy, with new remodelling spaces opening, but failing to undergo resorption when the next dose of denosumab is provided. The continuing increase in bone density with prolonged therapy raises the possibility of increases in bone strength and enhanced fracture prevention. BMD and fracture incidence will continue to be assessed as the phase 3 clinical trial continues further into its 7-year extension period (46).

### Pharmacotherapy and the 2010 OC Clinical Practice Guidelines

According to the 2010 OC Clinical Practice Guidelines, currently available pharmacotherapy reduces the relative risk of vertebral fractures by 30–70%, depending on the agent and the level of adherence (14). Despite a range of dosing frequencies and administration routes, the Guidelines cite consistent evidence from randomized clinical trials, suggesting that currently available treatments reduce vertebral fracture risk in postmenopausal women with osteoporosis (14).

Given the wide range of effective osteoporosis treatments, failure to identify and treat individuals at risk of fracture represents a significant missed opportunity to reduce morbidity and mortality. Unfortunately, many Canadian physicians do not routinely screen peri- and postmenopausal women for fracture history or assess patients’ 10-year fracture risk per OC Clinical Practice Guidelines, and they may be over-reliant on bone density measurements to assess risk (14,21). The Canadian Association of Radiology and Osteoporosis Canada (CAROC) tool, recommended by the OC Clinical Practice Guidelines, is an effective, validated approach to risk assessment. This tool incorporates a variety of patient data to evaluate fracture risk, including femoral neck BMD and patient age, along with fracture history and glucocorticoid use (14). The World Health Organization’s FRAX® tool, based on femoral neck BMD and other factors, likewise offers quantitative assessment of Canadian patients’ 10-year fracture risk (2).

## Conclusion

Addressing osteoporotic bone loss and resulting structural damage reduces risk of fractures and associated mortality, morbidity and cost of care. As the OC Clinical Practice Guidelines emphasise, effective risk assessment, with prompt introduction of pharmacotherapy to patients at high risk, are key steps in fracture prevention (14). Fortunately, Canadian physicians have a variety of effective therapeutics at their disposal. Understanding the bone remodelling pathways may be helpful in selecting appropriate treatment for patients and will be essential as new therapies continue to be introduced.

Denosumab is the newest of the first-line osteoporosis treatments and is distinguished from other antiresorptives by its novel mechanism of action and its twice-yearly dosing. Denosumab has begun to play an important role in the primary care of postmenopausal osteoporosis, as clinical data confirm that it leads to significant increases in BMD, with decreased risk of vertebral, hip and non-vertebral fracture (22,44,46–48).

## References

[1] Rizzoli R, Yasothan U, Kirkpatrick P (2010). Denosumab. Nat Rev Drug Discov.

[2] Kanis JA, Johansson H, Oden A, McCloskey EV (2009). Assessment of fracture risk. Eur J Radiol.

[3] Hussar DA, Stevenson T (2010). New drugs: denosumab, dienogest/estradiol valerate, and polidocanol. J Am Pharm Assoc.

[4] Papaioannou A, Kennedy CC, Ioannidis G (2009). The impact of incident fractures on health-related quality of life: 5 years of data from the Canadian Multicentre Osteoporosis Study. Osteoporos Int.

[5] Papaioannou A, Joseph L, Ioannidis G (2005). Risk factors associated with incident clinical vertebral and nonvertebral fractures in postmenopausal women: the Canadian Multicentre Osteoporosis Study (CaMos). Osteoporos Int.

[6] Perez-Lopez FR (2007). Vitamin D and its implications for musculoskeletal health in women: an update. Maturitas.

[7] Belavic JM (2011). Denosumab (Prolia): a new option in the treatment of osteoporosis. Nurse Pract.

[8] Brown JP, Fortier M (2006). Canadian Consensus Conference on Osteoporosis, 2006 Update. J Obstet Gynec Canada.

[9] Goltzman D (2002). Discoveries, drugs and skeletal disorders. Nat Rev Drug Discov.

[10] MacLean C, Newberry S, Maglione M (2008). Systematic review: comparative effectiveness of treatments to prevent fractures in men and women with low bone density or osteoporosis. Ann Intern Med.

[11] (2011). Prolia Product Monograph.

[12] Lipton A, Goessl C (2011). Clinical development of anti-RANKL therapies for treatment and prevention of bone metastasis. Bone.

[13] Bone HG, Bolognese MA, Yuen CK (2011). Effects of denosumab treatment and discontinuation on bone mineral density and bone turnover markers in postmenopausal women with low bone mass. J Clin Endocrinol Metab.

[14] Papaioannou A, Morin S, Cheung AM (2010). 2010 clinical practice guidelines for the diagnosis and management of osteoporosis in Canada: summary. CMAJ Canadian Medical Association Journal.

[15] Papaioannou A, Morin S, Cheung AM http://www.osteoporosis.ca/multimedia/pdf/Osteoporosis_Guidelines_2010_Background_And_Technical_Report.pdf.

[16] Osteoporosis Society of Canada (2005). Ontario’s strategy paves the way for better care across Canada. Osteoporos Update.

[17] Leslie WD, O’Donnell S, Lagace C (2010). Population-based Canadian hip fracture rates with international comparisons. Osteoporos Int.

[18] Adachi JD, Adami S, Gehlbach S (2010). Impact of prevalent fractures on quality of life: baseline results from the global longitudinal study of osteoporosis in women. Mayo Clin Proc.

[19] Bolland MJ, Grey AB, Gamble GD, Reid IR (2010). Effect of osteoporosis treatment on mortality: a meta-analysis. J Clin Endocrinol Metab.

[20] Hiligsmann M, Rabenda V, Bruyere O, Reginster JY (2010). The clinical and economic burden of non-adherence with oral bisphosphonates in osteoporotic patients. Health Policy.

[21] Bessette L, Ste-Marie LG, Jean S (2008). The care gap in diagnosis and treatment of women with a fragility fracture. Osteoporos Int.

[22] Miller PD, Bolognese MA, Lewiecki EM (2008). Effect of denosumab on bone density and turnover in postmenopausal women with low bone mass after long-term continued, discontinued, and restarting of therapy: a randomized blinded phase 2 clinical trial. Bone.

[23] Brown JP, Josse RG (2002). 2002 clinical practice guidelines for the diagnosis and management of osteoporosis in Canada. CMAJ.

[24] Seeman E (2003). Reduced bone formation and increased bone resorption: rational targets for the treatment of osteoporosis. Osteoporos Int.

[25] Manolagas SC (2000). Birth and death of bone cells: basic regulatory mechanisms and implications for the pathogenesis and treatment of osteoporosis. Endocr Rev.

[26] Weitzmann MN, Pacifici R (2006). Estrogen deficiency and bone loss: an inflammatory tale. J Clin Invest.

[27] Rosen CJ, Bilezikian JP (2001). Anabolic Therapy for Osteoporosis. J Clin Endocrinol Metab.

[28] Pallan S, Khan A (2011). Primary hyperparathyroidism: Update on presentation, diagnosis, and management in primary care. Can Fam Physician.

[29] Hanley DA, Watson PM, Hodsman AB, Dempster DM, Bilezikian JP, Raisz LG, Martin TJ (2008). Pharmacologic Mechanisms of Therapeutics: Parathyroid Hormone. Principles of Bone Biology.

[30] File E, Deal C (2009). Clinical update on teriparatide. Curr Rheumatol Rep.

[31] Baron R, Ferrari S, Russell RG (2011). Denosumab and bisphosphonates: different mechanisms of action and effects. Bone.

[32] Marie PJ, Kassem M (2011). Osteoblasts in osteoporosis: past, emerging, and future anabolic targets. Eur J Endocrinol.

[33] Cranney Ae G, Willan A, Griffith L (2002). Meta-analyses of therapies for postmenopausal osteoporosis. II. Meta-analysis of alendronate for the treatment of postmenopausal women. Endocr Rev.

[34] Russell RGG, Watts NB, Ebetino FH, Rogers MJ (2008). Mechanisms of action of bisphosphonates: similarities and differences and their potential influence on clinical efficacy. Osteoporos Int.

[35] Russell RG, Rogers MJ (1999). Bisphosphonates: from the laboratory to the clinic and back again. Bone.

[36] Ettinger B, Black DM, Mitlak BH (1999). Reduction of vertebral fracture risk in postmenopausal women with osteoporosis treated with raloxifene: results from a 3-year randomized clinical trial. Multiple Outcomes of Raloxifene Evaluation (MORE) Investigators. JAMA.

[37] (2006). Apo-Raloxifene Product Monograph.

[38] Yan MZ, Xu Y, Gong YX (2010). Raloxifene inhibits bone loss and improves bone strength through an OPG-independent mechanism. Endocrine.

[39] Delmas PD (2002). Treatment of postmenopausal osteoporosis. Lancet.

[40] PEPI Trial Writing Group (1996). Effects of hormone therapy on bone mineral density: results from the postmenopausal estrogen/progestin interventions (PEPI) trial. The Writing Group for the PEPI. JAMA.

[41] Moen MD, Keam SJ (2011). Denosumab: a review of its use in the treatment of postmenopausal osteoporosis. Drugs Aging.

[42] Lewiecki EM (2010). Treatment of osteoporosis with denosumab. Maturitas.

[43] Papapoulos S, Chapurlat R, Libanati C (2012). Five years of denosumab exposure in women with postmenopausal osteoporosis: Results from the first two years of the FREEDOM extension. J Bone Miner Res.

[44] Brown JP, Prince RL, Deal C (2009). Comparison of the effect of denosumab and alendronate on BMD and biochemical markers of bone turnover in postmenopausal women with low bone mass: a randomized, blinded, phase 3 trial. J Bone Miner Res.

[45] Seeman E, Delmas PD, Hanley DA (2010). Microarchitectural deterioration of cortical and trabecular bone: differing effects of denosumab and alendronate. J Bone Miner Res.

[46] Dore RK (2012). Data from extension trials: denosumab and zoledronic acid. Curr Osteoporos Rep.

[47] Kendler DL, Roux C, Benhamou CL (2010). Effects of denosumab on bone mineral density and bone turnover in postmenopausal women transitioning from alendronate therapy. J Bone Miner Res.

[48] Cummings SR, San Martin J, McClung MR (2009). Denosumab for prevention of fractures in postmenopausal women with osteoporosis. [Erratum appears in N Engl J Med. 2009 Nov 5;361(19):1914]. N Engl J Med.

[49] (2010). Fosamax Product Monograph.

[50] Black DM, Cummings SR, Karpf DB (1996). Randomised trial of effect of alendronate on risk of fracture in women with existing vertebral fractures. Fracture Intervention Trial Research Group. Lancet.

[51] Cummings SR, Black DM, Thompson DE (1998). Effect of alendronate on risk of fracture in women with low bone density but without vertebral fractures: results from the Fracture Intervention Trial. JAMA.

[52] (2011). Actonel Product Monograph.

[53] Harris ST, Watts NB, Genant HK (1999). Effects of risedronate treatment on vertebral and nonvertebral fractures in women with postmenopausal osteoporosis: a randomized controlled trial. Vertebral Efficacy With Risedronate Therapy (VERT) Study Group. JAMA.

[54] McClung MR, Geusens P, Miller PD (2001). Effect of risedronate on the risk of hip fracture in elderly women. Hip Intervention Program Study Group. N Engl J Med.

[55] (2011). Aclasta Product Monograph.

[56] Black DM, Delmas PD, Eastell R (2007). Once-yearly zoledronic acid for treatment of postmenopausal osteoporosis. N Engl J Med.

[57] Rossouw JE, Anderson GL, Prentice RL (2002). Risks and benefits of estrogen plus progestin in healthy postmenopausal women: principal results from the Women’s Health Initiative randomized controlled trial. JAMA.

[58] (2009). Forteo Product Monograph.

[59] Neer RM, Arnaud CD, Zanchetta JR (2001). Effect of parathyroid hormone (1–34) on fractures and bone mineral density in postmenopausal women with osteoporosis. N Engl J Med.

